# A Randomized Study on the Effect of Metformin Combined with Intensive-Exercise Diet Therapy on Glucose and Lipid Metabolism and Islet Function in Patients with Renal Cell Carcinoma and Diabetes

**DOI:** 10.1155/2022/7383745

**Published:** 2022-07-15

**Authors:** Yang Liu, Ling-Ling Meng, Jian-Wei Li, Yin-Shan Jin, Rui-Hua An

**Affiliations:** ^1^Department of Urology, The First Affiliated Hospital of Harbin Medical University, No.23 You Zheng Street, Harbin 150001, Heilongjiang, China; ^2^The Second Department of Urology, Cangzhou Central Hospital of Hebei Province, Cangzhou, Hebei, China; ^3^The Third Department of Endocrinology, Cangzhou Central Hospital of Hebei Province, Cangzhou, Hebei, China

## Abstract

**Objective:**

To evaluate the effect of metformin combined with intensive-exercise diet therapy on glucose and lipid metabolism and islet function in diabetes patients with localized renal cell carcinoma after laparoscopic resection.

**Methods:**

A total of 120 renal cancer patients with diabetes mellitus treated in the oncology department of our hospital from January 2018 to December 2020 were recruited and assigned via random number table method at a ratio of 1 : 1 to receive either metformin (control group) or metformin plus intensive exercise diet therapy (study group) after laparoscopic nephrectomy. Outcome measures included glucose and lipid metabolism, pancreatic islet function, lifestyle, clinical efficacy, and adverse reactions.

**Results:**

After the intervention, the fasting blood glucose (FBG), 2 h postprandial blood glucose (PBG), glycosylated hemoglobin (HbA1c), triglyceride (TG), total cholesterol (TC), and low-density lipoprotein cholesterol (LDL-C) of the two groups of patients decreased significantly, and the study group had significantly lower results. After treatment, the two groups had elevated levels of high-density lipoprotein cholesterol (HDL-C), fasting serum insulin (FINS), and homeostasis model assessment of *β*-cell function (HOMA-*β*), and higher results were obtained in the study group (*P* < 0.05). After the intervention, the study group showed higher results of health promoting lifestyle profile-II (HPLP-II) and a 12-month progression-free survival rate than the control group. There were no significant differences in the incidence of adverse reactions between the two groups.

**Conclusion:**

Metformin combined with intensive-exercise diet therapy significantly improves the glucose and lipid metabolism and islet function of renal cancer patients with diabetes and effectively enhances the 12-month progression-free survival. Further trials are, however, required prior to clinical application.

## 1. Introduction

Both malignant tumors and diabetes show a rising incidence in recent years, which poses a threat to the life safety of people. Research has reported a higher incidence of tumor progression in diabetic patients than in the nondiabetic population due to their impaired immune function [1]. Hyperglycemia provides energy for the tumor cells and promotes tumor growth [2]. Additionally, glucose and lipid metabolism disorders, anti-apoptosis of insulin, and mitosis enhancement in diabetic patients further induce the development of tumors [3]. A prior study reported that patients with fasting blood glucose levels higher than 7.8 mmol/l have a 25% higher chance of dying from tumors than those with a fasting blood glucose level lower than 5.6 mmol/l [4]. The renal cancer ranks the third in urinary system tumors, and its incidence has shown a rising tend [5]. A previous study revealed that the incidence and mortality of diabetes patients with renal cancer increased by 47% and 43% [6] versus those without diabetes. Diabetic complications such as hypertension and end-stage renal disease are associated with a high risk of renal cancer [7]. Therefore, the exploration of appropriate glucose control drugs for patients with renal cell carcinoma complicated with diabetes mellitus is of great significance.

Metformin is a widely used oral hypoglycemic agent and a first-line drug for type 2 diabetes. It activates AMP-activated protein kinase (AMPK) by activating liver kinase B1 (LKB1) to reduce blood glucose and blood insulin levels [8]. Research has demonstrated that metformin has an impact on the tumor suppressor gene liver kinase B1 (LBK1) to upregulate the expression of AMPK signal pathway and achieve the antitumor effect [9]. It has also been reported that metformin has obtained promising results in treating malignant tumors such as lung cancer, breast cancer, and kidney cancer [10]. A variety of traditional Chinese medicines have been proved effective in tumor treatment, such as Melissa officinalis L. and anthraquinone derivatives emodin [11–13]. An epidemiological study suggested that excessive intake of food, overnutrition, and lack of exercise are risk factors for impaired glucose tolerance and diabetes [14]. The importance of these approaches has been recognized with the development of therapeutic approaches and nursing concepts. The control of blood sugar and amelioration of the quality of life can be realized by the exercise diet therapy, yet its application in patients with renal carcinoma complicated with diabetes mellitus has been marginally explored. Accordingly, this study adopted metformin combined with exercise and diet therapy for patients with renal cancer and diabetes and gained desirable outcomes. The results are reported as follows.

## 2. Data and Methods

### 2.1. General Information

Totally, 120 cases of renal cell carcinoma with diabetes treated in the oncology department of our hospital from January 2018 to December 2020 were recruited and randomized into a control group (*n* = 60) and a study group (*n* = 60) using the random number table method. This study was reviewed and approved by the ethics committee of the First Affiliated Hospital of Harbin Medical University (approved no. LC 2018-11/233). The experiment was carried out in accordance with the Declaration of Helsinki, and all patients and their families provided undersigned informed consent forms.

### 2.2. Inclusion and Exclusion Criteria

#### 2.2.1. Inclusion Criteria

Patients aged 18~75 years, who were diagnosed with renal cancer by postoperative pathology after laparoscopic nephrectomy, who met the diagnostic criteria for type 2 diabetes, with a tumor stage T_1_N_0_M_0_ or T_2_N_0_M_0_, and with unilateral renal carcinoma with good contralateral renal function were included.

#### 2.2.2. Exclusion Criteria

Patients with a history of abdominal surgery; with serious dysfunction of the heart, liver, lung, and other organs; with complicated with hypertension or endocrine diseases; with sports contraindication, being unable to exercise as planned; and with mental and neurological dysfunctions that prevent cooperation with dietary interventions were excluded.

### 2.3. Intervention Methods

All patients were given conventional treatment after laparoscopic nephrectomy, and different blood glucose control schemes were adopted as follows.

#### 2.3.1. Control Group

The control group received oral administration of 0.5 g metformin (Zhengda Tianqing Pharmaceutical Group Co., Ltd., approval no. H20031104) for blood glucose control after meals, twice daily. The dosage was adjusted according to blood sugar and urine sugar, with the maximum daily dose within 2 g. Patients received exercise diet instruction, such as total calories control, diet instruction, a daylong stream of mini meals, and daily exercises. A similar metformin regimen was introduced to the patients in the study group.

#### 2.3.2. Study Group

The study group also received intensive exercise diet therapy: (1) diet intervention. The patients were instructed to follow an individualized diet plan based on their conditions, with carbohydrates accounting for 50% to 60% of total calories, protein about 10% to 20%, and fat about 20% to 30%. The diet was customized according to the patient's situation. (2) Exercise intervention: exercise stress electrocardiogram test combined with plasma lactate determination was used to determine the personalized target heart rate of moderate-intensity aerobic exercise. Patients were instructed by well-trained and experienced doctors to perform the exercise. A heart rate monitor was used to analyze the exercise heart rate and determine the exercise intensity to maintain the target heart rate. Patients were instructed to perform exercise 30 to 45 minutes each time, 6 times a week. Telephone follow-ups were conducted once every 2 weeks, and patients were reviewed once a month for diet and exercise instruction and intervention plan adjustment according to the patient's condition.

### 2.4. Outcome Measures

#### 2.4.1. Glucose and Lipid Metabolism and Islet Function

Before the intervention and at 6 months of intervention, fasting venous blood was collected to determine the patient's glucose and lipid metabolism indices, including fasting blood-glucose (FBG) and glycosylated hemoglobin (HbA1c), 2 h postprandial blood glucose (PBG), triglyceride (TG), total cholesterol (TC), high-density lipoprotein cholesterol (HDL-C), and low density lipoprotein cholesterol (LDL-C). Before the intervention and 6 months after the intervention, fasting serum insulin (FINS) was determined by high-performance liquid chromatography, and the homeostasis model assessment of *β*-cell function (HOMA-*β*) index was calculated by the following formula: [HOMA − *β* = 20 FINS/(FPG − 3.5)].

#### 2.4.2. The Quality of Life

The health-promoting lifestyle profile-II (HPLP-II) [15] was used to assess the quality of life before and after treatment, including health education, self-realization, interpersonal support, and nutrition enhancement. There are 48 items in 6 domains of regular exercise and stress management. Each item is divided into 1 to 4 points, which represent never, occasionally, often, and always. The higher the score, the better the quality of life.

#### 2.4.3. Long-Term Efficacy

All patients were followed up for 12 months after surgery, and the progression-free survival rate and overall survival rate of the patients were recorded.

#### 2.4.4. Adverse Reactions

The postoperative adverse reactions were recorded as per the Common Adverse Reaction Event Evaluation Criteria (CTCAE) [16].

Grade 1: mild and no symptoms that require no treatment. Grade 2: moderate symptoms that require minor, local, or noninvasive treatment, with restriction in daily activities. Grade 3: severe but not immediately life-threatening symptoms or disability that require hospitalization or prolonged hospital stay with restricted daily activities. Grade 4: life-threatening symptoms that require urgent treatment. Level 5: death due to adverse events.

### 2.5. Statistical Analysis

SPSS 23.0 was used for data analyses, and GraphPad Prism 8.0 was used to plot the graphics in the text. In this study, measurement data are expressed as mean ± standard deviation ($\bar{x}\pm \text{s}$), paired *t*-test was used for intra-group comparison between different timepoints, and two-independent sample *t*-test was used for intergroup comparison. Enumeration data are expressed by rate and analyzed using the chi-square test. *α* = 0.05 was assumed as statistical significance.

## 3. Results

### 3.1. Comparison of General Data

As shown in Table 1, the basic data of the two groups of patients, such as age, gender, BMI, tumor stage, and tumor diameter and location, were comparable (all *P* > 0.05).

### 3.2. Comparison of Glucose Metabolism Indicators

**Figure 1:**
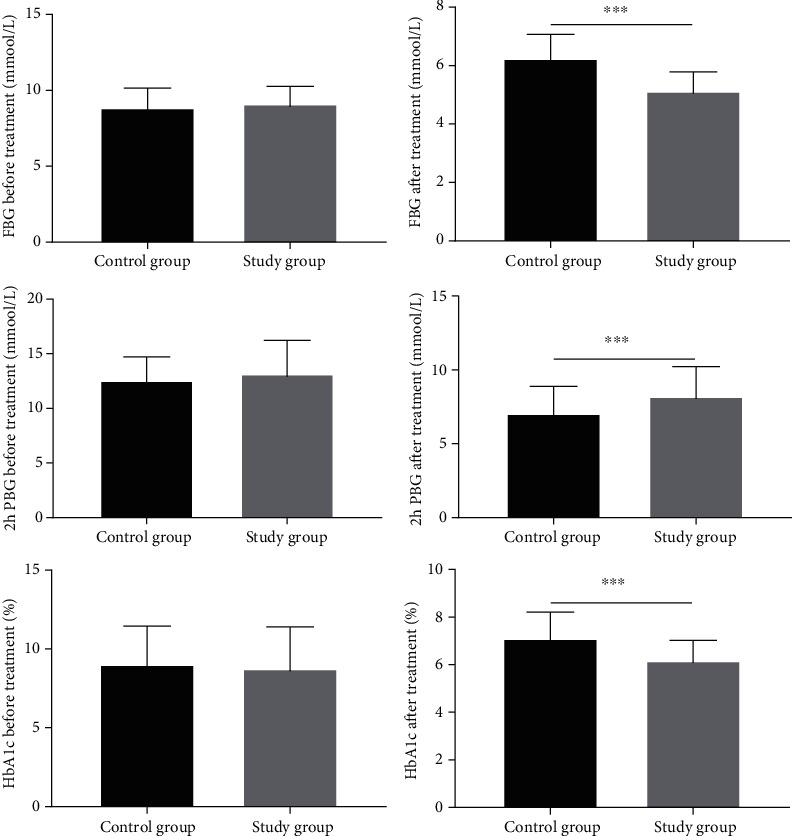
Comparison of the glucose metabolism levels. Note: ^∗∗∗^ indicated *P* < 0.001 compared between the two groups.

As shown in Figure 1, there were no significant differences in FBG, 2 h PBG, and HbA1c between the two groups of patients before the treatment (all *P* > 0.05). After the treatment, the above indices of the two groups of patients were significantly decreased, and the study group had significantly lower results (all *P* < 0.05).

### 3.3. Comparison of Blood Lipid Indexes

**Figure 2:**
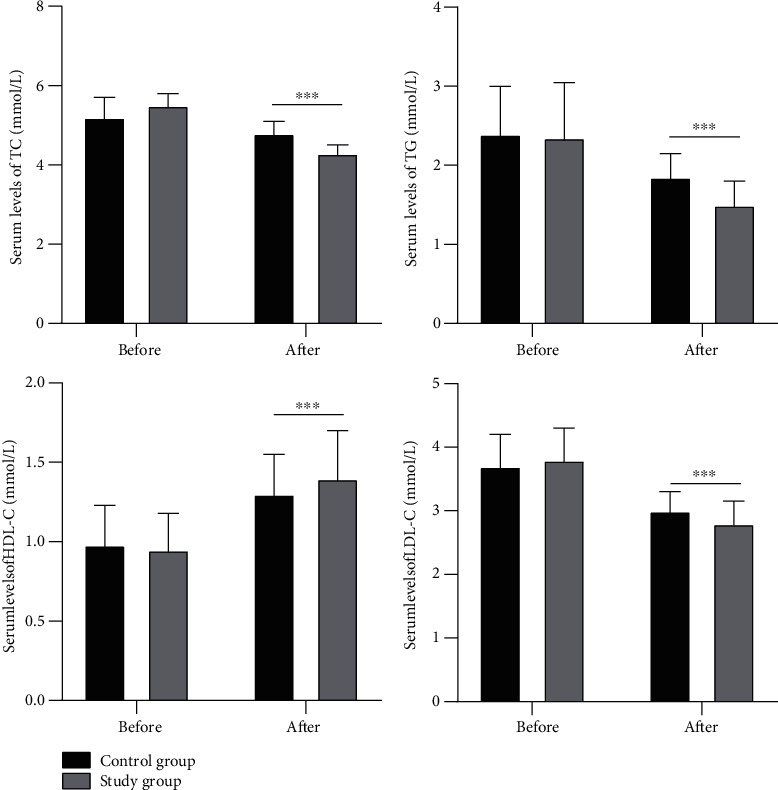
Comparison of the serum levels of TC, TG, HDL-C, and LDL-C. Note: ^∗∗∗^ indicated *P* < 0.001 compared between the two groups.

As shown in Figure 2, before the treatment, there were no significant differences in serum TC, TG, HDL-C, and LDL-C levels between the two groups of patients (all *P* > 0.05). After the treatment, TC, TG, and LDL-C of the two groups of patients decreased significantly, and the study group had lower results. The level of HDL-C was significantly increased, and the study group showed significantly higher results than the control group (all *P* < 0.05).

### 3.4. Comparison of Islet Function

As shown in Table 2, before the treatment, there were no statistically significant differences in FINS and HOMA-*β* between the two groups of patients (all *P* > 0.05). After the treatment, the above indicators of the two groups of patients were elevated, in which the study group had significantly higher results than the control group (all *P* < 0.05).

### 3.5. Comparison of Lifestyles

**Figure 3:**
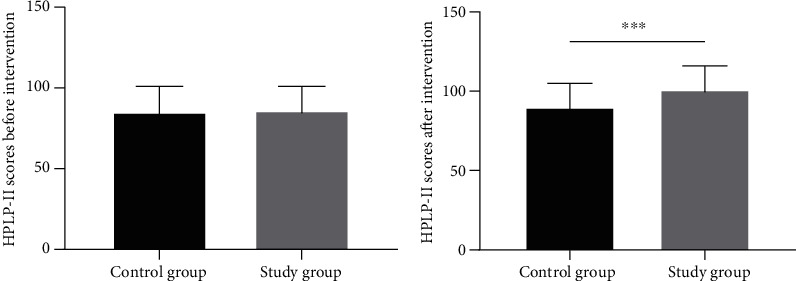
Comparison of the HPLP-II scores. Note: ^∗∗∗^ indicated *P* < 0.001 compared between the two groups.

As shown in Figure 3, there were no significant differences in HPLP-II scores between the two groups of patients before the treatment (*P* > 0.05). After the treatment, the study group had higher levels of HPLP-II than the control group (*P* < 0.05).

### 3.6. Comparison of the Long-Term Efficacy

As shown in Table 3, 6 months after treatment, there were no significant differences in the progression-free survival rate and overall survival rate of the two groups of patients (all *P* > 0.05). 12 months after treatment, the progression-free survival rate of the study group was significantly higher than that of the control group (*P* < 0.05). The two groups presented similar overall survival rate (*P* > 0.05).

### 3.7. Comparison of Adverse Reactions between the Two Groups of Patients

As shown in Table 4, there were no significant differences in the incidence of all grades of adverse reactions and ≥3 grades of adverse reactions between the two groups of patients (all *P* > 0.05).

## 4. Discussion

In recent years, the incidence of kidney cancer has been increasing year by year. Despite the rapid development of early detection in recent years, the survival of renal cancer patients remains unsatisfactory [17]. Research has shown that diabetes is a risk factor for the poor prognosis of kidney cancer, which necessitates timely treatment and regular monitoring of the blood glucose level of patients with kidney cancer [18]. Diabetes is a chronic metabolic disease with abnormally elevated blood sugar levels as the main manifestation, among which noninsulin-dependent diabetes (type 2 diabetes) accounts for more than 90%. The main mechanism is insulin resistance. The body's ability to absorb and utilize glucose decreases, leading to elevated blood sugar levels. Disorders of glucose metabolism may trigger various pulmonary dysfunctions and involve multiple organs and tissues such as cardiovascular system, cerebrovascular system, eyes, liver, and kidney, which seriously compromises the quality of life of patients [19]. Research has shown that hyperinsulinemia and elevated insulin-like growth factor-1 in patients with type 2 diabetes are closely related to malignant tumors. About 8-18% of new malignant tumors cases are comorbid with diabetes [20]. Surgery is the treatment of choice for localized renal cancer, with a 5-year disease-free survival rate of about 90% and a recurrence and metastasis rate of 20-30% [21].

Metformin effectively lowers the blood sugar and lipids of patients and is considered the optimal choice for the treatment of type 2 diabetes. It has been found that metformin could lower the risk of prostate cancer, breast cancer, liver cancer, and other tumors in diabetic patients [22]. Lifestyle changes, high-sugar, high-fat diet intake, and long-term nutritional imbalance are the main causes of impaired glucose tolerance and insulin resistance [23]. Therefore, in addition to drug treatment, exercise diet intervention contributes to the control of blood sugar levels. In the present study, the glucose and lipid metabolism and insulin function of the two groups of patients were significantly improved after the treatment. For patients with kidney cancer and diabetes, the role of dietary exercise intervention is essential in blood sugar control and disease recovery. In the present study, metformin combined with intensive-exercise diet therapy was adopted for the first time and obtained good clinical effect. However, this study still has the following shortcomings. First, the effect of intensive-exercise diet therapy in this study is easily affected by cognitive ability and compliance, which complicates the evaluation of the implementation of dietary exercise programs. Secondly, intensified exercise diet intervention requires community family care support, along with high medical costs, which may prevent its extensive application. In the future, the new treatment concepts need to be popularized, and multicenter, large-scale studies need to be conducted to obtain more reliable data for clinical references.

## 5. Conclusion

The use of metformin combined with intensive-exercise diet therapy significantly improves the glucose and lipid metabolism and islet function of renal cancer patients with diabetes and effectively increases the 12-month progression-free survival rate. Further trails are, however, required prior to clinical application.

## Figures and Tables

**Table 1:** Comparison of the general data.

	Control group (*n* = 60)	Study group (*n* = 60)	*t*/*χ*^2^	*P*
Age ($\bar{x}\pm \text{s}$, year-old)	59.28 ± 8.46	61.50 ± 11.25	1.220	0.225
Gender (male/female)	38/22	34/26	0.556	0.456
BMI ($\bar{x}\pm \text{s}$, kg/m^2^)	28.59 ± 5.16	27.13 ± 4.92	1.576	0.118
Staging and grading			0.926	0.336
T_1_N_0_M_0_	42	37		
T_2_N_0_M_0_	18	23		
Tumor diameter	3.26 ± 1.06	3.11 ± 0.82	0.815	0.417
Tumor location			0.906	0.341
Left	36	41		
Right	24	19		

**Table 2:** Comparison of the pancreas islet function ($\bar{x}\pm \text{s}$).

	FINS (mU/l)			HOMA-*β*		
	Before	After	*Δ*	Before	After	*Δ*
Control group (*n* = 60)	8.12 ± 2.15	11.26 ± 2.86	1.24 ± 0.53	3.94 ± 1.05	5.79 ± 1.25	1.60 ± 0.63
Study group (*n* = 60)	8.06 ± 2.51	9.22 ± 1.47	0.56 ± 0.27	4.12 ± 1.18	4.72 ± 1.18	0.51 ± 0.25
*t*	0.141	4.917	8.855	-0.887	4.846	12.46
*P*	0.888	<0.001	<0.001	0.377	<0.001	<0.001

**Table 3:** Comparison of the long-term clinical outcome.

	6 months after intervention		12 months after intervention	
	PFS	OS	PFS	OS
Control group (*n* = 60)	51	56	40	51
Study group (*n* = 60)	53	58	51	55
*χ* ^2^	0.289	0.175	5.502	1.294
*P*	0.591	0.675	0.019	0.255

**Table 4:** Comparison of all grade AEs grade ≥ 3 AEs.

	All grades	Grade ≥ 3
Control group (*n* = 60)	22	12
Study group (*n* = 60)	16	9
*χ* ^2^	1.386	0.520
*P*	0.239	0.471

## Data Availability

The datasets used during the present study are available from the corresponding author upon reasonable request.
